# The Incidence and Prevalence of Dementia in Alberta, Canada between 2014 and 2022

**DOI:** 10.1002/alz70860_099037

**Published:** 2025-12-23

**Authors:** Mohammad Z I Chowdhury, Julia G Kirkham, Eric E. Smith, Zahra Goodarzi, Gavin Thomas, Susan E Bronskill, Julie G Kosteniuk, Dallas Seitz

**Affiliations:** ^1^ Alberta Health Services, Calgary, AB, Canada; ^2^ University of Calgary, Calgary, AB, Canada; ^3^ Hotchkiss Brain Institute, University of Calgary, Calgary, AB, Canada; ^4^ ICES, Toronto, ON, Canada; ^5^ Sunnybrook Research Institute, Toronto, ON, Canada; ^6^ Dalla Lana School of Public Health, University of Toronto, Toronto, ON, Canada; ^7^ University of Saskatchewan, Saskatoon, SK, Canada

## Abstract

**Background:**

The number of people affected by dementia in Canada is anticipated to increase significantly in coming years. The province of Alberta is expected to have the largest relative increase in the number of people affected by dementia among all provinces in Canada. We determined the prevalence and incidence of diagnosed all‐cause dementia in Alberta, Canada between 2014 and 2022 using population‐based health data.

**Methods:**

Population‐based administrative health data were used to identify all individuals ages 65 years and older who were diagnosed with dementia between 2014 and 2022 in Alberta, Canada. A validated case ascertainment algorithm was used to identify cases of dementia. The prevalence of all‐cause dementia was reported for all individuals on April 1, of each year. Annual numbers of incident cases of dementia were reported on April 1 for each year using individuals newly diagnosed with dementia in the preceding year.

**Results:**

The overall prevalence of dementia in Alberta increased from 30,050 in 2014 to 43,802 in 2022 (45% relative increase). The number of incident cases of dementia increased from 6,884 individuals in 2014 to 8,741 in 2022 (27% relative increase). The incidence rate of dementia declined during this time period from 1.48/100 person years in 2014 to 1.37/100 person years in 2022 (*p* <0.001).

**Conclusion:**

The number of prevalent and incident cases of dementia increased significantly in Alberta between 2014 and 2022 although the incidence rate of dementia is decreasing during this time. This information has important implications for the planning supports and services for this population.

